# American Medical Association

**Published:** 1858-05

**Authors:** 


### EDITORIAL.

### AMERICAN MEDICAL ASSOCIATION.

We have just received the first day's proceedings of this body, together with the annual address of President P. F. Eve, of Nashville, and as it contains a succinct account of the history of its proceedings up to the present time, we give place to it in our editorial columns.

The officers elect are—for President, Dr. Harvey Lindsley, of Wash., D. C.; Vice-Presidents, Drs. W. L. Sutton, of Ky., Thos. O. Edwards, of Iowa, Josiah Crosby, of N. H., W. C. Warren, N. C.

### ADDRESS.

Gentlemen of the American Medical Association,—We meet under most auspicious circumstances, and have been welcomed to the most favorable position ever occupied by our profession on this continent. The very ground on which we stand may be considered sacred; has been set apart from a common to a special purpose, and is national. Invited as we have been to this magnificent temple, furnished and dedicated by a generous foreigner to science; in the presence of that towering monument, designed to commemorate the worth of him ever enshrined first in the hearts of his countrymen; surrounded by the glorious recollections constantly associated with this government; and before the great men and assembled wisdom of the nation; it becomes us to discharge the important duties which have called us together with honor to ourselves and benefit to our profession. Inspired by its benevolent spirit, and invoking the aid of an ever present and omnipotent God to preside over our deliberations, we may here renew our professional obligations, learn to love each other better, and resolve henceforth to be more faithful to our high vocation, that its dignity may be maintained and its usefulness extended.

Knowing as I do full well the value of time in our short sessions, and how much is expected from this meeting, the half hour set apart for this customary address will be restricted to subjects appropriate to the occasion. From this stand point in the history of our meetings, it is proper to recall what has already been achieved, that we may be better prepared profitably to engage in the labor now awaiting our deliberations. This summary of our transactions is the more necessary, since, by a disastrous fire in 1851, the first four volumes of our proceedings have been destroyed, and are of course inaccessible to all new members, the last report of the commitee on publication having announced the fact that not one complete set of them was on sale.

The grand object of a convention of the physicians of the United States, held the previous year in the city of New York, was carried into effect in Philadelphia, May, 1847, by organizing this association; and just ten years ago, the first general assembly met in Baltimore. Since then, annual meetings have been convened in our large cities for the transaction of business, and the proceedings regularly published each year. Ten large octavo volumes now comprise the transactions of the American Medical Association, being the contributions of its two thousand members delegated to represent the medical institutions of thirty States and Territories.

As set forth in convention, the ultimate purposes of this body are to cultivate and advance medical knowledge; to elevate the standard of medical education; to promote the usefulness, honor, and interests of the medical profession; and collaterally to enlighten and direct public opinion in regard to the duties, responsibilities, and requirements of medical men; to excite and encourage emulation and concert of action in the profession, and to facilitate and foster friendly intercourse between those engaged in it.

In carrying forward these desirable changes, embracing as they do medical science, medical education, and medical ethics, no one believes that we have done everything demanded for the good of the profession, or that all our great designs could have been attained in the brief space of ten years. The work assumed by the association, it was well known, would take time, labor, and united efforts. It comprehended higher requisitions for admission into a learned profession; prescribed the course of instruction; demanded a separation in the teaching and licensing power; proposed a code to regulate the intercourse between physicians, their patients, and the public; and claimed that every one within its pale should assiduously cultivate the science of medicine and promote its best interests. And however extensive or radical may have been these contemplated plans, still on the whole it can safely be assumed that the American Medical Association has been no failure.

It has advanced medical knowledge, and promoted the usefulness of the medical profession. There will be found in the ten volumes of its printed transactions the results of the meetings held in Baltimore, Boston, Cincinnati, Charleston, Richmond, New York, St. Louis, Philadelphia, Detroit, and Nashville, that no less than three hundred pages are devoted to medical education; over five hundred to hygiene, including the sanitary condition of many of our large cities; six hundred to botany and indigenous plants; one hundred and fifty to obstetrics; four hundred to medical literature; seven hundred and fifty to medical science proper; more than a thousand to surgery; and two thousand to practical medicine, including the epidemics and prevalent diseases of nearly every State in the Union.

Special reports have been made from committees appointed for the purpose, on the effects of anæsthetic agents, ether and chloroform; on the influence of tea and coffee on the diet of children and the laboring classes; on the supposed influence of the cerebellum over the sexual propensities; the results of operations for the cure of cancer; the introduction of water and gas into cities; two reports on the blending and conversion of types of fever; the action of water on lead pipes and the diseases proceeding from it; affections of the uterus; a nomenclature of diseases adapted to the United States, having reference to a general registration of deaths; the sources of typhus fever and the means of their extinction; the permanent cure of reducible hernia; the topical use of water in surgery; the agency of refrigeration by radiation of heat as a cause of disease; the results of surgical operations in malignant diseases; the acute and chronic diseases of the neck of the uterus; the nature of typhoid fever; coxalgia or hip-joint disease; the treatment of morbid growths within the larynx; the sympathetic nerve in reflex phenomena; the medical and toxicological properties of the cryptogamic plants of the United States; erysipelas; the influence of the hygrometrical state of the atmosphere on health; the diet of the sick; pathology, causes, symptoms, and treatment of scrofula; the preservation of milk; the effects of alcoholic liquors in health and diseases; hydrophobia; the changes in milk produced by menstruation and pregnancy; the sanitary police of cities; treatment of cholera infantum; use and effects of nitrate of silver applied to the throat; strychnine; infant mortality in large cities, the sources of its increase and means of its diminution; medico-legal duties of coroners; new principle of diagnosis in dislocation at the shoulder-joint; the flora, fauna, and medical topography of Washington Territory; the nervous system in febrile diseases, etc., etc.

Prizes have been awarded by the association to the authors of the following essays, viz: On the corpus luteum of menstruation and pregnancy, for 1851.

On the variation of pitch in percussion and respiratory sounds in physical diagnosis, for 1852.

On the cell, its physiology, pathology, and philosophy.

And on the surgical treatment of certain fibrous tumors of the uterus, heretofore considered beyond the resources of art, for 1853.

On a new method of treating ununited fractures and certain deformities of the osseous system, for 1854.

On the statistics of placenta prævia, for 1855.

On the physiology and chief pathological relations of the arterial circulation, for 1856.

On the excito-secretory system of nerves, its relations to physiology and pathology.

And on experimental researches in relation to the nutritive value and physiological effects of albumen, starch, and gum, when singly and exclusively used as food, for 1857.

Carefully prepared reports have been published by the association of the various epidemics and diseases which have prevailed during the past ten years throughout our widely-extended country, and the mortuary statistics and public health of our large cities minutely ascertained. Charts, maps, diagrams, tables, and plates have been freely employed to illustrate these subjects, so important to the general welfare of the people. Every State and Territory, every large city and sick community, with scarcely an exception, has had its hygienic condition explored by this body; and dysentery and cholera, typhoid and yellow fevers, have specially claimed the attention of our members. The communications on deformities after fractures, found in our eighth, ninth, and tenth volumes, constitute the basis of the best monograph ever issued from the press. This work, it may be predicted, will do more than all others to check the reckless and speculative spirit of suits for mal-practice against medical men; for in addition to teaching a useful lesson to the profession in the prognosis of fractures, its testimony is so conclusive in reference to the usual results of these accidents, that judicial decisions must hereafter be regulated by it.

Besides these contributions to medical knowledge, this association has taken action to prevent the importation into our country of "worthless, adulterated, and misnamed drugs, medicines, and chemical preparations," for which a member of the United States Senate has publicly declared that if we had accomplished nothing else, this alone should have entitled us to the gratitude of the nation; it recommended to the different States the adoption of a regular system of registration of births, marriages, and deaths; memorialized Congress to secure steerage passengers in our emigrant vessels medical attention, and due amount of space between decks; appointed a committee to ascertain the best means of preventing the introduction of disease by emigrants into our large cities; and considered many interesting individual cases.

This is a mere index to what the American Medical Association has done for medicine during the first ten years of its existence. A simple reference to the professional facts spread out upon its pages, is abundant and satisfactory proof how extensive, varied, and valuable are its contributions to medical science, and its ten volumes an overwhelming and congratulatory answer to the taunting proclamation of the Edinburgh Review of 1820, "What does the world yet owe to American physicians and surgeons?" In September, 1854, the editors of the Association Medical Journal of Great Britain published our code, and declared that this body of physicians was the most enlightened representatives of the greatest medical constituency in the world, of which it would be presumptuous in them to speak in terms of praise. They said of the volumes of the *Transactions* already published, that the duties of the standing committees have been ably and thoroughly performed; that the progress of medical science as a whole, its prominent divisions into practical medicine, surgery, and obstetrics, carefully and accurately traced in a series of reports worthy of the highest praise, had been reported in a clear, concise, and comprehensive manner, reflecting the highest credit upon the committees, and also upon the association in selecting them for their respective duties; and in regard to what has been done in the laborious investigation of the indigenous medical flora of the Union; examination into and reports upon the adulteration of drugs; sanitary condition of the various States, and difference between them in the public health; the study of epidemics and of special scientific subjects; the committees, continue these European medical authors, have collected and published a vast amount of highly valuable information. They moreover assert their belief that our success, especially in ethical reform, depends solely in the moral power inseparable from a constitution based upon the principle of equal representation, which they affirm they not only greatly admire, but can scarcely refrain from envying.

Here, then, is a reply to the above invective pronounced against the medical profession of America, voluntarily called forth from the countrymen of its author, and before he had been in his grave ten years,* by the contributions of this body to medical science within seven years of its organization. Upon such disinterested evidence, such full, free, and candid confessions, and from such a source, may rest the claims of the American Medical Association for proofs of the benefits it has conferred on medicine. A most active and powerful agent in disseminating useful medical knowledge on this continent, it is highly probable that no similar institution has ever been more successful in carrying out its chief object

* Sidney Smith died in 1845.

—the promotion of science—than the one now assembled in this hall.

It has done something (perhaps, all it could under the circumstances) to elevate the standard of medical education. An influential motive calling forth this organization, was the proposed attempt to correct the defects in the plan of instruction and conferring the degree, then generally adopted in our medical colleges; and one of the first resolutions passed, even when the profession had assembled in convention, was the creation of a committee to report at an early day on these exciting subjects. Improvement in the system of teaching medicine, and a change in the power granting the diploma, if not reformation in the schools, have ever since agitated the profession, and consumed a considerable portion of the time of our sessions. The only power to control the economy of the colleges which this body possesses, is exclusively moral, advisory, or recommendatory, and not legislative or legal; and while it may be true that no set of resolutions, presented by the several committees, have been fully carried into effect, still it cannot be denied that important changes calculated to advance medical education, have nevertheless been made. At least seven professors compose the faculty in all our schools, the one or two exceptions to this being in those in which the science is taught nine consecutive months. Not less than a period of four full months' instruction now constitutes a course of lectures, and even this is exceeded in most of the institutions. But one annual course is now delivered with scarce an exception, and an interval is thus allowed for reading or private instruction. The association has clearly defined what shall be taught. It has inquired into the practical operation of all the colleges of the land; scrutinized the general condition of medical teaching in every State; compared it with that of the most enlightened nations; called attention to preliminary education, and declared what it ought to be; advised higher requisitions and a more rigid examination for obtaining the degree; and has, by its free discussions and oft reiterated expressions in regard to the business of teaching and regulating the schools, undoubtedly prevented greater abuses. It has never ceased to urge at every meeting, the pressing necessity for a more thorough preparation, and greater attainments in candidates for the honors of the profession.

This subject, gentlemen, is one upon which you will be called to take action. A committee chosen at Nashville, is to report here on medical education. It is composed of gentlemen from different sections, who, while familiar with the systems of teaching medicine in our country, are yet disconnected from all the colleges. It would seem to be a desirable object to settle at this meeting the future relation of the schools to this association. Our sessions then might become less educational in character, and hereafter more scientific. And at the present stage of our proceedings, after all that has been said and done on the subject, the time has surely arrived for a decision. I cannot believe the colleges have any interested motives before this body; they of all others should be the last to oppose a more thorough cultivation of medicine, and ought, by such a course to become unworthy of their trust, and unwelcome members of a great national congress of physicians, whose grand design is to promote medical science. We have now reached a period in our history, when this voluntary association is to determine what medical organizations, be they State, county, or city societies, hospitals, boards, or schools, are entitled to be represented in its meetings. It alone can, of course, prescribe the requisitions for its own delegates. If created to improve and advance medical education (and this is in accordance with its own expressed declarations), then it is quite certain the schools must be controlled. It has but to speak on this point and it will be obeyed, for it is now too late for any physician to oppose, or any medical college to set at defiance the moral power of this body.

As to the first object of an ethical nature over which the association designed to exert its influence, that of enlightening and directing public opinion in respect to the duties, responsibilities, and requirements of medical men, we are free to confess little or nothing has been done. Nor is there much probability that any great change will soon, if ever, be effected. The work itself, in the very nature of things, is utopian. How is it possible to enlighten or direct the public mind on the economy of a science which it practically denies to exist? We ought to recollect that the time has not long passed since grave professors in our colleges signed certificates recommending nostrums; or what was done even last year in London, at Middlesex Hospital, by its regular surgical staff. These reminiscences, however unpleasant, may serve somewhat to moderate our indignation against those who would insult the profession, or who entertain a very low estimate of the scientific acquirements of physicians, even at the present day. The profession must first fully comprehend its duties and responsibilities, and the proper and special qualifications for the practice of medicine, before any attempt can succeed to get the public to appreciate what these are, or acknowledge the ethical impropriety of employing secret remedies. If we make no distinction between the regular and irregular practitioner, between the physician and the proprietor of a nostrum, we are alone censurable that two such opposite characters are so generally confounded by the community. Until we are more honest, more united, truer to ourselves and our calling, and cultivate a proper *esprit du corps*, in vain is it to expect a change in public opinion regarding medical science. To prevent disease or relieve the sick is a most benevolent and honorable vocation, and when one conceals for selfish ends a valuable medicine, he ceases to be honest and is void of philanthropy; for, by attempting to place a moneyed valuation upon pain and life, he becomes a trader in human physical sufferings; he estimates in dollars and cents the groans and tears of his fellow-creatures. He may profess what he pleases, but his piety is not of the Bible, and has not a jot or tittle of Christianity about it, for that teaches us to love our neighbors as ourselves. Eschewing politics, and seeking no aid from State or Church, we should become a law unto ourselves, or rather act above all law save the divine, since it is quite certain we alone must protect the honor of the medical profession. And thank God, standing this day, the proudest of my life, before this goodly assembly, and at the capital of our common country, I can announce that here, to the American Medical Association, it may with safety be forever confided. By its recent acts, proclaimed throughout the length and breadth of this wide domain, this body has denounced all fellowship with irregular practices, and erected a barrier impassable to honor and respectability.

Having learned wisdom from a more careful examination of the statistics and results of deformities after fractures, the question occurs if we have not ourselves unwittingly made patients expect too much from remedial agents. Disease in itself is a destructive process, which we can only prevent or relieve, and as, of course, we cannot create or restore, should we not, therefore, be more chary of the little word, "cure?" The monument erected to Ambrose Pare, the father of surgery, bears the modest inscription, in reference to the wounds he treated, "*Je les pansay et Dieu les guarit.*" * Empirics may boast that they cure, and doctors of divinity may sustain them, but the physician knows it is God who healeth all our diseases.

On that branch of ethics which relates to ourselves—that of encouraging emulation and concert of action among physicians, and fostering friendly intercourse in the profession—the association has been eminently successful. It has far exceeded the most sanguine expectations in overcoming all opposition; in creating an admirable code, now adopted everywhere; in or-

---

* Ancient French.

ganizing state, county, and city societies; in bringing together physicians from the remotest parts of our immense territory; in awaking the whole profession to its true interests; and in blending us into a common harmonious fraternity. Without law or authority, but by moral suasion have we been united as one man, and possess this day the power to be felt over this entire continent. There never has been a more propitious period for medicine in America; never greater evidence of vitality and extended usefulness in our ancient and benevolent calling; never better feeling or more confidence of success than now by our united effort to do good in the great cause of suffering humanity.

We have seen, gentlemen, how much this association has achieved in its infancy to elevate honorable medicine. A wide field for scientific investigation is before us; much territory still remains to be redeemed; the wilderness is yet to blossom as the rose, and the leaves to be gathered for the healing of nations. The hygienic condition of the nation, of such immense interest to our people—that first and important question ever before the profession, the prevention of disease—is to be improved. We are to search after truth, and when found, it is to be generously applied for the good of mankind. The work is a self sacrificing and benevolent one, but it is grand and sublime, even God-like; for it has to do with pain and disease, life and death; and we rejoice to know that, whenever or wherever called upon, the members of our profession and of this association have never failed in any duty, and have been faithful to the end. Yea, many of them have stood alone between the living and the dead, and cheerfully laid down their lives to stay the pestilence and destroyer.

The very waters at our feet, as they sweep onwards to the ocean, pass in sight of a city where three years ago no less than four-fifths of our profession in that community, swelled, too, as their ranks had been by volunteers from this body, fell manfully contending with disease and death; and on a late occasion, when one of our steam-packets, having been injured by a collision, went down in an instant, carrying every soul on board into the depths of the ocean, among the passengers was a member of this association. To the inquiry where was he during the heart-rending scenes of a sinking ship, freighted with human lives, promptly came the affecting and sublime eulogy from one who knew him well, that so long as a woman or child remained unprovided for, he* never left the ill-fated

* Prof. Carter P. Johnson, of Richmond, Virginia.

Arctic. How near akin was his gallant spirit to that of him, who, during a subsequent and similar occurrence, after seeing every woman and child committed to his care safely rescued from his foundering bark, after sending the last parting message to his family, and discharging every duty without one lingering ray of hope, calmly assumed his commanding position on the deck of his vessel, and as she glided from under him into the yawning billows, instinctively uncovered to meet his fate and his God. While the wild waves are sighing a requiem over the unseen burying places of these illustrious dead, the benedictions of a grateful people are continually ascending over the forty graves of the martyred heroes of Norfolk. These were our companions, who died in the noble service of that calling to promote the best interests of which has assembled us together.

Gentlemen of the American Medical Association, we have convened for important purposes; great events are before us; the interests of humanity are here; the hopes of the profession are in this meeting; the eyes of the medical world are upon us. May we then so act in view of surrounding circumstances, that "The skill of the physician shall lift up his head; and in the sight of great men he shall be in admiration."

